# The many faces of SRPK1†

**DOI:** 10.1002/path.4846

**Published:** 2017-02-01

**Authors:** Nicholas Bullock, Sebastian Oltean

**Affiliations:** ^1^ School of Physiology, Pharmacology and NeuroscienceUniversity of BristolBristolUK; ^2^ Department of SurgeryCardiff and Vale University Health BoardCardiffUK; ^3^ Bristol Renal, School of Clinical SciencesUniversity of BristolBristolUK

**Keywords:** cancer, NSCLC, stem cell, SRPK1, Wnt/β‐catenin signalling

## Abstract

Serine–arginine protein kinase 1 (SRPK1) phosphorylates proteins involved in the regulation of several mRNA‐processing pathways, including alternative splicing. SRPK1 has been recently reported to be overexpressed in multiple cancers, including prostate cancer, breast cancer, lung cancer, and glioma. Several studies have shown that inhibition of SRPK1 has anti‐tumoural effects, and SRPK1 has therefore become a new candidate for targeted therapies. Interestingly, in terms of molecular mechanism, SRPK1 seems to act heterogeneously, and has been reported to affect several processes in different cancers, e.g. angiogenesis in prostate and colon cancer, apoptosis in breast and colon cancer, and migration in breast cancer. A recent report adds to this puzzle, showing that the main effect of SRPK1 overexpression in non‐small‐cell lung carcinoma is to stimulate a stem cell‐like phenotype. This pleiotropy might be related to preferential activation of different downstream signalling pathways by SRPK1 in various cancers. © 2016 The Authors. *The Journal of Pathology* published by John Wiley & Sons Ltd on behalf of Pathological Society of Great Britain and Ireland.

## Abstract 2

Linked Article: Gong et al. J Pathol 2016; 240: 184‐196.

The transformation of a normal human cell into cancer is a highly complex process that involves the accumulation of mutations alongside a plethora of complex non‐genetic mechanisms. It is now understood that these processes culminate in the development of a heterogeneous mix of genetically distinct subclones that, together with endothelial, stromal and various other infiltrating cell types, collectively make up a tumour 1, 2. Further evaluation of the hierarchical organization of several tumour types has led to the identification of a subpopulation of self‐renewing cells that show stem‐cell like properties, the so‐called cancer stem cells (CSCs), which are thought to be important in the development of tumour recurrence, metastasis and resistance to therapy 3.

Serine–arginine protein kinase 1 (SRPK1) is a protein kinase that specifically phosphorylates proteins containing serine–arginine‐rich (SR) domains. SR proteins are involved in regulating several RNA‐processing pathways, including RNA stability, alternative splicing, and translation 4. Both SRPK1 and its downstream targets have been shown to be involved in a number of biological and pathological processes, with increased expression being seen in a range of cancer types, including breast, colon, pancreas and prostate cancer 5, 6, 7. In their recent article, Gong *et al* 8 explored the role of SRPK1 in the promotion of a CSC‐like phenotype in human non‐small‐cell lung carcinoma (NSCLC).

First, Gong *et al* 8 demonstrated that *SRPK1* mRNA levels are elevated in NSCLC patients as compared with normal individuals, with expression being increased in NSCLC lines relative to that of normal lung epithelial cells and non‐cancerous adjacent tissues. They went on to use immunohistochemistry to evaluate relative expression levels of SRPK1 in human NSCLC tissues. Statistical analyses demonstrated SRPK1 to be strongly associated with clinical stage and TNM classification, with high levels being linked to shorter overall survival than in patients with low‐level expression. This association remained true when samples grouped according to clinical stage were compared; this, when combined with SRPK1 expression being an independent prognostic factor in multivariate analyses, led the authors to assert that SRPK1 levels may be useful as a prognostic indicator in NSCLC 8.

CSCs have been implicated in the development of drug resistance in NSCLC 9. Therefore, the authors undertook a series of well‐designed *in vitro* experiments exploring the potential role of SRPK1 in the promotion of the CSC phenotype. They found that *SRPK1*‐transduced cells expressed higher levels of pluripotency‐associated markers, and showed Hoescht33342 dye exclusion (a marker for cancer stem cells) and greater self‐renewal capacity 8. The use of *SRPK1*‐specific RNA interference oligonucleotides, which suppress expression of the protein, demonstrated the converse effect, thus suggesting that SRPK1 promotes stem cell accumulation and adoption of a stem cell‐like phenotype. A supporting set of *in vivo* experiments on BALB/c nude mice showed that tumours formed by *SRPK1*‐transduced NSCLC cells were larger than those formed by controls, with the opposite being observed when *SRPK1*‐silenced cells were inoculated.

Finally, through the use of gene set enrichment analysis, a luciferase reporter assay for T‐cell factor/lymphoid enhancer factor transcriptional activity, immunofluorescence, and polymerase chain reaction of downstream gene targets, Gong *et al* 8 sought to determine whether the underlying mechanisms of stem cell phenotype development are linked with SRPK1 action on the Wnt–β‐catenin pathways, which have previously been implicated in the development and activity regulation of CSCs. Upregulation of SRPK1 led to both increased beta‐catenin translocation to the nucleus and increased transcriptional activity in NSCLC cell lines, leading them to conclude that it is through activation of this signalling pathway that SRPK1 exerts its effect on the promotion of stemness 8.

## SRPK1 in other cancers: mechanistic differences

**Figure 1:**
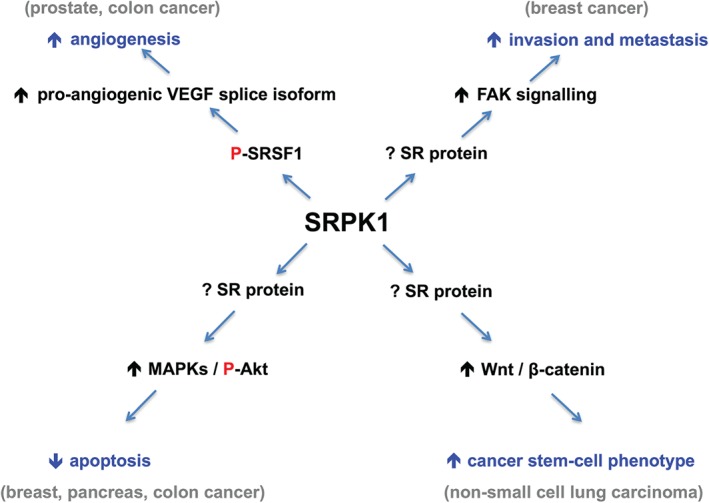
Examples of pleiotropic effects of SRPK1 in various cancers. Depending on the type of cancer, SRPK1 regulates different cellular properties. Akt, protein kinase B; FAK, focal adhesion kinase; MAPK, mitogen‐activated protein kinase. P denotes phosphorylation.

Not only does this article implicate SRPK1 in yet another cancer type, but the findings are of particular interest because they demonstrate an alternative mechanism by which SRPK1 exerts its tumourigenic effects. Much of the previous research on SRPK1 has focused on its role in the regulation of pre‐mRNA splicing. SRPK1 contains a PKc superfamily kinase domain, and is able to phosphorylate SR proteins, thereby regulating their functional activity. These proteins have been shown to play an important role in alternative pre‐mRNA splicing, as well as other steps of RNA metabolism, such as polymerase II transcription, nuclear export of mature mRNA, and nonsense‐mediated mRNA decay 10, 11. The archetypal member of the SR protein family and a key substrate of SRPK1 is serine–arginine‐rich splicing factor 1 (SRSF1), a proto‐oncogene that appears to exert its tumorigenic effects via a number of mechanisms, particularly alternative pre‐mRNA splicing 10. One pre‐mRNA splicing event for which SRSF1 has been shown to be a key regulator is that of vascular endothelial growth factor A (VEGF‐A) (a regulator of angiogenesis) to form pro‐angiogenic and anti‐angiogenic isoforms 12. In our recently published study, we found that increased SRPK1–SRSF1 axis activity in prostate cancer led to preferential splicing of pro‐angiogenic VEGF‐A mRNA, thereby implicating modulation of angiogenesis as an important mechanism in the pathogenesis of this cancer subtype 6 (Figure 1).

Interestingly, although SRPK1 expression also appears to be increased in breast cancer 5, contemporary work seems to suggest an alternative mechanism of pathogenesis in this tumour type. In their recent study, van Roosmalen *et al* 13 found that SRPK1 expression correlated with preferential metastasis of breast cancer to the lungs and brain. Subsequent SRPK1 knockdown appeared to suppress metastasis to distant organs, and to inhibit focal adhesion reorganization, suggesting that its activity and function are fundamental to tumour cell migration in this particular cancer type 13. Additionally, work published by Lin *et al* also suggests that SRPK1 may exert some of its tumourigenic effects in breast cancer through RNA‐binding motif protein 4‐mediated reduction in the expression of pro‐apoptotic *IR‐B* and *MCL‐1_S_* transcripts, thereby modulating sensitivity to apoptotic signals 14.

The conclusion of the Gong *et al* study that SRPK1 promotes the development of a stem cell‐like phenotype in NSCLC suggests yet another mechanism of SRPK1 action in this cancer type 8. Interestingly, although the authors assert that promotion of this phenotype may be due to SRPK1‐mediated activation of Wnt–β‐catenin pathways, the exact mechanism that drives the activation remains to be elucidated. One possibility, as suggested by extrapolation of the findings of a recent study 15, is that SRPK1 upregulation may lead to increased phosphorylation of SR proteins such as SRSF1, which in turn recruit β‐catenin mRNA and enhance its translation in an mTOR‐dependent manner. Although, to date, there have been no specific in‐depth studies of the relationship between SRPK1 and angiogenesis in lung cancer, these findings may indicate that SRPK1 preferentially drives a particular pattern of gene expression that is dependent on cancer type, thereby leading to a different predominant phenotypic picture in each. It is possible that SRPK1 activates different downstream signalling pathways in different cancers, thereby resulting in various cellular processes being affected (Figure 1). This effect may be due to phosphorylation of different SR proteins, which may be tissue‐specific and cell‐specific in each cancer.

Interestingly, a recent study has revealed a complex relationship between SRPK1 and another kinase, CDC2‐like kinase 1 (CLK1) [a member of the CDC2‐like kinase (CLK) family], in the regulation of SR protein phosphorylation 16. Unlike the serine–arginine protein kinases such as SRPK1, which are only able to phosphorylate Arg–Ser dipeptides, the CLKs are localized strictly within the nucleus, and are able to phosphorylate both Arg–Ser and Ser–Pro dipeptides. A symbiotic relationship between SRPK1 and CLK1 has been demonstrated, with binding of SRPK1 to an RS‐like domain in the N‐terminus of CLK1 facilitating release of phosphorylated SR proteins, and hence subsequent splice‐site recognition and spliceosome assembly 16. With this relationship in mind, it would be interesting to investigate whether CLK1 levels and/or activity vary across different cancer types and consequently modulate SRPK1 function.

## SRPK1 as a therapeutic target in cancer

As well as its potential use as a biomarker, the emerging evidence supporting the role of SRPK1 in the pathogenesis of several cancers, irrespective of its underlying mechanism, makes it a possible and indeed attractive therapeutic target. Although more research is needed to fully elucidate the biology of SRPK1 and its downstream target pathways, early data concerning the potential advantages of its inhibition seem encouraging. For example, in a therapeutic proof‐of‐principle experiment in the field of prostate cancer, we found that the SRPK1 inhibitors were able to inhibit phosphorylation of SR proteins and switch VEGF‐A splicing *in vitro* 6. Furthermore, we demonstrated that one of the agents, SPHINX, decreased tumour growth when administered intraperitoneally to a mouse model of orthotopic prostate cancer.

It is hoped that the publication of further high‐quality studies, such as the one presented by Gong *et al* 8, will allow us to gain a more in‐depth understanding of the mechanisms underlying the apparent role of SPRK1 in cancer biology. In doing so, they may provide the basis for ongoing drug development and, eventually, clinical trials. Furthermore, if indeed the mechanistic differences between tumour types persist as more studies emerge, we may see variation in the clinical role of small‐molecule SRPK1 inhibitors, depending on cancer type. For example, perhaps they may be used to stave off metastases in patients with early breast cancer, and be used as anti‐angiogenic therapy in prostate cancer (Figure 1).

### Author contributions statement

NB, SO: responsible for the writing and revision of the manuscript.
